# Relationships between work-family conflict and family structure in the lives of working mothers in Hungary – a pilot study

**DOI:** 10.1186/s40359-024-01925-0

**Published:** 2024-08-06

**Authors:** Barbara Sztányi-Szekér, Ágnes Hőgye-Nagy, Anita Szemán-Nagy

**Affiliations:** 1 Doctoral School of Human Sciences, Doctoral Program on Psychology, University of Debrecen, Debrecen, Hungary; 2 Department of Work and Social Psychology, Institute of Psychology, University of Debrecen, Debrecen, Hungary; 3 Department of Personality and Clinical Psychology, Institute of Psychology, University of Debrecen, Debrecen, Hungary

**Keywords:** Family structure, Work-family conflicts, Work and family involvement, Work and family life satisfaction, Working mothers

## Abstract

**Background:**

The family, as the basic socialization environment, is a complex dynamic system that - as a whole and through its subsystems - is in relationships with other social systems (Bagdy in Family socialization and personality disorders. Nemzeti Tankönyvkiadó, Budapest, 2002; Lakatos et al. in Mentálhigiéné és Pszichoszomatika 21(1):56–85, 2020). The system with which the family system has long-term relationships is the work system/environment. Creating and maintaining a work-life balance has become a central issue in our societies, as they are two of the most organising forces, and reconciling them is a very difficult task due to the demands and expectations coming from both directions, often simultaneously (Makra et al. in Magyar Pszichológiai Szemle 67(3):491–518, 2012). This kind of “double burden” primarily affects women, but their increasing role in the labour market is not necessarily followed by an equal sharing of work within family life (Engler et al. in Work-life balance in women’s careers. In: Tardos K, Paksi V, Fábri Gy (eds) Scientific careers in the early 21st century. Belvedere Meridionale, Szeged, pp 114–126, 2021). We hypothesise that involvement in work negatively correlates with work-life balance, making it more difficult to integrate into the family. It was expected that the relationship between the number of children and mothers’ professional involvement would be negative. A positive correlation was expected between the age of the youngest child and the mothers’ work involvement. On the other hand, a family united by cohesion and resilience leads to higher job satisfaction.

**Methods:**

For the present analysis, we analysed the relationships between work-family conflict and family structure in working mothers with children in a sample of 273 participants. The self-reported questionnaire included demographic data and 2 standard questionnaires: the Work-Family Conflict Questionnaire and the Olson-Family Test (FACES-IV.). The study was conducted in Hungary.

**Results:**

No significant relationship was found between work involvement and work-family conflict. A negative relationship was observed between work involvement and family involvement. Similiarily, no significant relationship was found between the number of children, the age of the youngest child and work involvement, contrary to expectations. The findings indicate a positive relationship between good family cohesion, flexibility and job satisfaction.

**Conclusion:**

Striking a work-family balance is a challenging process for families with young children, especially working mothers. A mutually negative relationship between work and family involvement has been shown. The importance of a well-functioning family, with adequate cohesion and flexibility, is reflected in family and job satisfaction. The relationship between work-to-family conflict and job involvement is moderated significantly only when family flexibility is low. The results from the present pilot study indicate important relationships between variables and point to further research directions worth investigating in a larger sample in the future.

**Supplementary Information:**

The online version contains supplementary material available at 10.1186/s40359-024-01925-0.

## Background of the study

### Primary socialisation agent - the family system

The family “institutional system”, structured in different forms, has existed in all cultures for thousands of years. It is in the family unit, which is regarded as the primary socialising unit, that the initial stage of becoming human and adult takes place, followed by the subsequent stages of friendship, work, and many other small groups. The sense of belonging to the family community contributes to the formation of family structure and family identity, including family functions and role divisions. Nowadays, in addition to traditional family role divisions, there are also specific transformations in family life, involving internal structures and role merging [1]. In addition to values related to communities and family, members of today’s society are increasingly characterised by a kind of individualistic “tendency” that encourages people to prioritise their individual well-being, desires, and goals, giving greater emphasis to individual preferences [2]. In the second half of the 20th century and in the first decade of the 21st century, the issue of factors influencing changes in marriage forms and the structure and functioning of families aroused the interest of many researchers. Great attention was paid to changing the employment status of women and increasing their participation in the labor market. Economic factors have also had a significant impact, mostly a negative one, on the “propensity” to get married, the time of marriage and the willingness to have children [3–6]. In addition to the traditional family model, there are more and more alternative ways of living, which also means more freedom for people, as the choice between them is more flexible. These choices are becoming more and more accepted, leading to the emergence of new family forms [7]. Thus, families today can be perceived as many different forms and varieties, and there are many different ties of cohabitation or relationships. For example, in addition to nuclear families in marriage, there are long-lasting relationships following divorce, or remarriage, but it is also important to mention non-marital forms of life, “patchwork” families, or more commonly known as mosaic families, or single parents raising their child/children alone, widows, long-distance relationships and virtual relationships. The increasingly widespread non-family variety of the 21st century, such as “singleness” is also worth mentioning [8]. According to the interactional view, the family is actually an open, dynamic system in a state of dynamic balance: in the event of any deviation, large or small, from the state of balance, the system will constantly strive to restore itself. The family can be an optimal system, functioning properly at its optimum, and be quite strong and resilient, but it can also be a dysfunctional system. Maintaining this stability through constant change is part of how systems work. This is called primary change. In contrast, if the nature of the system itself changes, for example when it starts to operate in a new state of balance, this is secondary change. It is important to note that primary changes always occur within the family system, while secondary changes occur in family life cycle changes. In many cases, families may experience a standstill and even a crisis as they struggle to find effective ways of dealing with these situations [9]. The process of achieving a state of balance provides a good understanding of phenomena such as family interactions, where individuals relate to each other through different behaviours. The relationships between family members influence each other, controlling the individuals involved [10]. Family members’ behaviours are influenced implicitly rather than by rules that are often spoken to each other, but they do not necessarily dictate what will happen, as families can change and continue to do so [9]. In connection with these changes, the definition of the family life cycle is an important concept in the life of families, which is actually a theoretical framework of how a family functions under different cultural and social expectations [10]. The two fundamental missions of families are to provide opportunities for change and stability, the latter provides the basis for a sense of security, while the former contributes greatly to family development. Family development can be characterised by gradualness through the different stages in the family life cycle [11]. During this process, it is normal for all families to experience conflicts and tensions, which means that periodic crises are unavoidable. Changes of varying strength and evoking various emotions - such as marriage, childbirth, divorce, illness, death, and problems at work, and others - affect the original pattern/structure of the family and, consequently, a new structure is formed. In these structural transformations, a new state of balance must be established, which the family should still be able to control in order to maintain continuity while functioning well [10]. All of these constructs appear together in the “circumplex model” of family structure theory, associated with David H. Olson, which measures and studies the dynamics “occurring” in families and couple relationships [12, 13]. The two most important variables of family and relationship dynamics can be illustrated graphically: cohesion (adherence, unity) and adaptability (flexibility, adjustability) within the family. The third facilitating dimension of the model is communication. The model focuses on how family members can function within this system in relation to each dimension. The dimension of cohesion (adherence, unity) represents the emotional relationships between family members and shows how individuals can harmonise their being together or separated. Based on the above, 4 levels of cohesion have been identified: disjointed, fragmented, cohesive, and embedded, of which those in the middle, i.e. the fragmented and cohesive type, are considered to be balanced, healthy families [14, 15]. The dimension of adaptivity (flexibility, adaptability) measures the balance between stability and change within the family, and shows whether family members are able to adapt to possible changes in the family structure and to the effects of sudden external stimuli (e.g., childbirth, job change, illness, etc.). With the right level of adaptability, the family structure can change, and the internal roles and rules can be adapted to the new situation. There are also 4 levels of adaptive capacity: rigid, structured, flexible, and chaotic types [14, 16, 17]. According to Olson [15], families that can be characterised by harmonious functioning also fall into the two middle dimensions (structured and flexible), i.e., those that can find and maintain a state of balance in terms of stability, security, and openness to change, which makes them more adaptable. According to the Circumplex Model, individuals and relationships can encounter difficulties when flexibility is either extremely high (chaotic) or extremely low (rigid) for a prolonged duration. Conversely, relationships that maintain a moderate level of flexibility (structured and flexible) are better suited to navigate the delicate balance between change and stability. While there is no definitive optimal level for every relationship, it is common for relationships to face challenges when they consistently operate at either end of the model (rigid or chaotic) in the long run [12].

The third dimension, communication, is considered a kind of background dimension in the model. Optimal and effective communication helps to ensure the interoperability between the dimensions of cohesion and adaptivity, and to establish and maintain the right balance. Research findings also confirm that balanced, well-functioning family systems and couple relationships are characterised by positive, meaningful communication [14–16].

Because of the above, in the context of the dimensions included in the model, it is possible to distinguish between well-functioning (balanced) and problematic families, which makes it possible to understand the reactions to stress and the adaptation skills and coping strategies at the individual, couple/spousal and family level [13].

### Changes in the role of women in the family and their situation in the labour market

When analysing the life and structure of families, a key issue is that of gender roles, which have a significant impact on the development of family structure, from the division of tasks within the household, partner relationships, cohesion, and communication, to conflict management and decision-making at different levels. The traditional division of roles between the two genders is expressed by the head of the family, to which position the man, partner/husband, father of the family was “assigned” primarily because of his function as the breadwinner of the family. This role included the supreme right of decision-making in family matters, taking responsibility for family members, representing the family to the outside world and society as a whole, and above all, ensuring the material resources mentioned above. In this traditional view of the family, working in the household and looking after and bringing up children were typically female roles [18]. The labour market has been restructured as a result of major socio-economic changes, which, beyond the individual level, also have a major impact on the life, internal relations, and structure of families. Consequently, gender roles in family life and at work are undoubtedly changing [18, 19]. In one of her most recent works, Nobel Prize winner Prof. Claudia Goldin mentions that, a century ago, it was natural for a woman with a degree to have to choose between family life and work/career. Today, there are more women with degrees than ever before, but they still face severe challenges at home and work. In her research on the subject, she sought to trace how, while gender equality has changed significantly, women of different generations still relate to the issue of work-life balance [20]. *Workplace productivity theory* suggests that mothers are less effective in their workplaces thus contributing less social capital to the economy, which may also be a consequence of the fact that household chores and childcare, which in many cases are still traditional female “tasks”, take so much energy away from women that they become less productive in the workplace [21]. In recent years, several studies have dealt with the phenomenon of the so-called *motherhood penalty* in the labour market, which is also closely linked to gender inequalities. Differences in earnings also highlight the vulnerability of women compared to men. According to analyses, women bringing up their child/children alone are more likely to become poor than men in the same situation [22]. Research also shows that despite changes in female employment and labour market participation, there has been no relevant reform in the division of tasks within the family, with the result that today’s working women have to carry double burdens and responsibilities [23]. Despite a clear upward trend in the number of female workers, as shown by statistical data, their employment rate is still persistently lower than that of men [5]. The “situation” of women has been the subject of many international and national studies, but the areas most frequently examined are the realisation of equal opportunities in the labour market, the issue of work-life balance, and the “reintegration” of women with children into the world of work and their opportunities in the workplace [5, 24, 25]. From a socio-economic point of view, therefore, women should have more children, based on current data, and should also be more present in the world of work than they are nowadays. However, it is an important question how working women with children perceive this problem on an individual level, how they themselves consider the harmony of work and family life, the question of the compatibility of the two areas, their role and importance in their lives [26].

### Work-life balance in the lives of women with children

There is a growing trend worldwide for the increased number of working mothers to become a catalyst for the phenomenon known as “work-family conflict” [27]. The vast majority of working parents complain of difficulties in achieving and maintaining a work-life balance and in meeting the demands and expectations of both. People try to meet the requirements in the best possible way, although they often conflict with each other, often in terms of fulfillment in time [25, 28, 29]. Based on the theory of limited resources, work-family conflict can be understood as a “struggle” between family and work for resources that are limited for individuals, such as energy, attention, and time [30]. Meanwhile, the individuals have to share resources between the work and family fields, where they face role demands from both directions that make it difficult, or at worst impossible, to meet the demands of one field while meeting the demands of the other. In effect, this will reduce resource inputs to the disadvantage of one role, and when different roles conflict, this can lead to role conflict, which can be a source of stress in people’s lives [31, 32]. Role conflict can lead to psychological and physical problems, resulting in emotional exhaustion, inadequate parental care, job dissatisfaction, and poor work performance [25, 33]. Furthermore, they have a negative impact on people’s psychological and mental well-being and consequently on life satisfaction [29, 34, 35]. On the issue of work-to-family conflict, it is important to distinguish between *work-to-family conflict* (work-related, work demands interfere with family life) and *family-to-work conflict* (family-derived, family demands disturb the work sphere) [25, 36]. Grönlund and Öunfound found, that longer working hours increase work-life conflict [37]. According to research carried out by Gatrell and associates [38], men and women experience work-family expectations differently, it is mostly women who take on a greater share of household and childcare tasks in addition to their work. There is evidence from research that this is more common in the case of women, considering that men have more stable working hours [39, 40]. Steiber makes a time-based and intensity-based difference between work-family conflict, with women being more likely to experience intensity-based conflict (due to the “double burden” problem), while men are more likely to experience time-based conflict [41]. Exploratory research by Makra et. al. also reveals direct effects on work-family interference: family involvement is increased by marital status and high childbearing, while overtime work is reduced; work involvement is negatively affected by family involvement [25]. In their research, Drummond and associates found that the support of a work supervisor and family/partner reduced the work-family conflict to a greater extent for women and contributed more to psychological well-being and satisfaction with family and life than for men [42]. According to the research carried out by Okonkwo, work-family conflict can be described as a negative spill-over, whereby problems in one area have a negative impact on the other (e.g., increasing job dissatisfaction leads to dissatisfaction in family life [43]. In contrast, Wilson and Wagner have analysed the phenomenon of positive spill-over from work-related dissatisfaction to family life [44]. Their research results confirmed their hypothesis that satisfaction in the everyday workplace had a positive impact on everyday spouse/partner relationship satisfaction and emotions in the family. The changes in traditional gender roles have contributed to the fact that women’s participation in the labour market is no longer seen as an option, but increasingly as a social demand [45]. In the increasingly widespread dual-earner family form, statistics show that men actually help more with housework and childcare, but generally do not even reach the same level as women, not even change the traditional gender division of labour in cases where women are the breadwinners [46]. The issue of work-life balance thus very often becomes a serious dilemma in the lives of families and in the daily lives of couples. Workloads and tasks often take time away from family or partners, which is a major source of stress for everyone [47].This lack of time, either from work to family or from family life to work, causes problems and difficulties for parents with young children, and overwhelmingly for mothers [48]. The results from a national survey [49] also show - in line with the global view - that the vast majority of respondents consider the balance between work and family life to be the biggest challenge in their lives. Both international and national literature emphasise that, as is the general view, professionals in management positions tend to treat the work-family balance as a ‘women’s issue’ [50, 51]. This view is supported by the results of several Hungarian studies on the subject, which show that women are most often responsible for the difficulties of reconciling work and home responsibilities [52, 53]. In contrast to the Western European trend, a significant proportion of Hungarian women put family first after starting a family and having children, and put individual goals on the back burner. This is also due to the fact that even today, traditional values, attitudes, and expectations are likely to have a significant influence [54–56].The issue of work-life balance, however, has a major impact not only on women’s individual and individual objectives, but also on those of society as a whole, as one of the major challenges facing our society is the phenomenon of aging. The shift in the time of starting a family and having children, the radical reduction in the number of children, and the difficulties of making a living are linked to the need to work. Once family life has begun, it becomes particularly difficult to reconcile work and private life [55].

### Purpose of the study

#### Aims and hypotheses

Socio-economic changes and modernisation have led to a significant increase in the employment rate of mothers with young children in the labour market worldwide. While balancing work and family roles is not an easy task and can cause conflict in family life, there is a growing body of research on the positive benefits of both. In recent years, researchers in this field have placed more emphasis on analysing the quality of work-family interactions [57, 58]. Concerning traditional workplace roles, the findings show evidence of greater involvement of women in family life [59]. As a result, women are faced with a double burden of being able to adequately perform the tasks required to balance work and family life [23]. In terms of the Hungarian specificities, according to the most recent data, Hungary’s population is steadily declining, with deaths increasingly outnumbering births [60]. Despite all these data, many measures are being taken to increase birth rates (homemaking allowance, mortgage remission, baby loan, education benefits for mothers, and family-friendly jobs). In Hungary, demographic issues are up to date at the moment. It seems that the series of subsidies are being implemented, so we tried to approach the primary issue from a different angle (work-life balance) and to examine how these women who work and have a child/toddler at the same time experience this situation, what their mental well-being is like, what their satisfaction indicators are, because, in addition to economic factors, these psychological factors are also significant.

In the present study, we aimed to investigate how those who are currently working and raising children can reconcile work and family life in the context of the conflicts arising from the various workloads and family ‘expectations’, and how this relates to some aspects of the family structure. We hypothesised a link between higher levels of work involvement and the prevalence of work-to-family conflict (the negative interference of work demands with family life). In addition, we assume a negative relationship between work and family involvement. We consider a higher number of children and lower age of the youngest child to be related to the disadvantages of the mother’s labour force participation. It is also assumed that individuals with higher levels of cohesion and flexibility in their own families are more satisfied with their family life and their work. We also wanted to investigate the possible moderating effect of family flexibility on relation to work-family involvement and work-to-family conflict and family-to-work conflict. The following hypotheses were formulated to test the relationships outlined in the bibliography:

##### H1

It is hypothesized that there is a relationship between work involvement and the frequency of family conflict arising from work.

##### H2

We assume that there is a negative relationship between work involvement and family involvement.

##### H3

We assume that there is a negative relationship between the number of children and the involvement of working women with children in work, and expect positive relationship between the age of the youngest child and the mother’s involvement in work; the more children there are in the family and the younger the children, the less these women can be involved in work.

##### H4

We assume that there is a positive relationship between adequate family cohesion and satisfaction with family life and work: women who have a more optimal level of family cohesion are more satisfied with their family life and work.

##### H5

We assume that there is a positive relationship between family flexibility, satisfaction with family life, and work: those who have a more optimal level of family flexibility are more satisfied with their family life and work.

##### H6

The relationship between family involvement and work-to-family conflict is moderated by family flexibility, just like the relationship between work involvement and family-to-work conflict.

## Methods

### Sample

The questionnaire package used in this study was completed by a total of *N* = 273 currently employed mothers with one or more children at the time of completion. The mean age of the study subjects was 40.5 years (SD = 6.4), the youngest respondent was 23 years old and the oldest was 55 years old. The highest proportion of respondents (58.6%) live in a county town or city (28.9%), followed by a rural municipality (5.9%), village (3.3%) or capital (3.3%). Concerning the number of children, the mean was estimated at 1.9 (SD = 0.798), with the fewest children in the family being 1 and the most being 5. The average age of the youngest child of the responding mothers was 8.2 (SD = 5.1), and there were cases where the responding mother also indicated the fetal age of the unborn child in the questionnaire. The oldest child in the sample was 18 years old on average. Most of the study subjects selected in the sample had two (48.7%) or one (32.2%) child, compared to mothers with three (16.5%), four (1.5%) and five (1.1%) children. In terms of marital status, the highest proportions are married (74.7%) or in a civil partnership (9.2%). A much smaller proportion lived as a couple (6.6%) or were single (5.9%) with a child/children and the smallest proportion were divorced (3.7%). Most of the mothers had a university degree (75.5%) or secondary school education (21.6%), with a few having a vocational school (2.6%) as the highest level of education. For unrelated respondents, a significant proportion of their partners also had a university (47.6%) or secondary school (26.4%) education, while less so for vocational school (15.8%) and 8 general schools (0.7%).

### Procedure

The questionnaires were available online in Google Forms, shared with the participants on the personal Facebook pages of the researchers, and distributed to the respondents using the snowball method (no one was contacted directly). The questionnaire bundle was completed anonymously, on a voluntary basis, and respondents were free to withdraw at any time. Subjects were first provided informed consent to meet the criteria: to investigate the relationship between family structure and work-family balance, we expected responses from mothers aged 18 and over who were currently working and had/have a child/ more children. Participation in the survey was preceded by a full survey description, providing respondents with detailed information on the purpose of the study and how to participate.

### Measuring instruments

Data from the following questionnaires included in the online questionnaire package were used for the analysis: a self-report questionnaire on demographic data (age, gender, relationship status, number and age of children) and 2 standard questionnaires (Work-Family Conflict Questionnaire and the Olson-Family Test (FACES-IV.)) [14, 25].

The Work-Family Conflict Questionnaire (25 items) was developed by Emese Makra, Dávid Farkas and Gábor Orosz (2012) with the aim of creating and validating a work-family conflict questionnaire, which consists of different parts to be applied to a Hungarian sample. The scale is composed of the following dimensions: work-to-family conflict, family-to-work conflict, work-family involvement, also life and job satisfaction. The questionnaire distinguishes between two versions of work-family conflict (five statements each): (a) family-related conflict coming from work (e.g., “My job often makes me cancel important family activities or events.“) and (b) work-related conflict coming from the family (e.g., “The tension in my family prevents me from fulfilling my work responsibilities.“). The scales used to assess involvement were based on two questionnaires, which also distinguish two types: family involvement, (e.g., “I am most concerned about things related to my family.“), and work involvement, (e.g., “My work is a very important part of my life.“). Both instruments contain five to five statements. The last dimension examined was satisfaction. Two versions of this were operationalised, also based on two questionnaires: job satisfaction (three statements, e.g. “Overall, I am very satisfied with my job”), and life satisfaction (five statements, e.g. “I am satisfied with my life”). For each question, subjects were asked to rate on a four-point Likert scale how typical the statements were of themselves (1: not at all typical of me, 2: not typical of me, 3: typical of me, 4: very typical of me). The authors used Cronbach’s alpha values to introduce the reliability of the questionnaire, which showed high values in all cases: work-to-family conflict (0.848), family-to-work conflict (0.821), work involvement (0.831), family involvement (0.855), life satisfaction (0.843) and work satisfaction (0.808) [25].

The Olson-Family Test (FACES-IV.) scale contains a total of 62 items and is composed of eight subscales, six of which measure members’ perceived family cohesion (cohesive, fragmented, embedded) and adaptivity (flexible, rigid, chaotic). Two additional subscales are satisfaction with family communication and satisfaction with family life. The scales for balanced and unbalanced cohesion and adaptivity consist of 7 items each. The scales measuring family communication and satisfaction with the family consist of 10 items each. Respondents are asked to think about the extent to which the items in the scale are specific to their current family. For each item, respondents can answer on a five-point Likert scale (1 = not at all typical of our family, 2 = less typical of our family, 3 = somewhat typical of our family, 4 = usually typical of our family, 5 = very typical of our family) for the scales cohesion (“Family members are involved in each other’s lives”), adaptivity (“Family members make rules together” and communication (“Family members listen to each other carefully”). For questions 53–62, where respondents are asked to answer to what extent they are satisfied with a particular area of family life (“. the cohesion between family members”), they are also asked to choose one of five response options, but here in the order 1 = very dissatisfied, 2 = somewhat dissatisfied, 3 = generally satisfied, 4 = very satisfied, 5 = completely satisfied). The higher scores a person has on each scale, the more typical the level of cohesion or adaptivity measured by the scale, the more effectively they communicate, and the more satisfied they are with family functioning [13, 17].

## Results

The analyses for the study were conducted using the SPSS statistical software. For reliability and descriptive statistics see Tables 1 and 2.

**Table 1:** The Cronbach’s alpha coefficients for the Hungarian version of the Olson-Family Test (FACES-IV.) and the Work-Family Conflict Questionnaire

	Cronbach’s alpha reliability			Cronbach’s alpha reliability	
Olson-Family Test (FACES IV.)	0.822	good			
Work-Family Conflict Questionnaire	0.725	acceptable	work-to-family_ conflict	0.851	good
			family-to-work_ conflict	0.836	good
			family_ involvement	0.682	doubtful
			work_involvement	0.805	good

In order to interpret the results, we considered it important to display the corresponding values related to descriptive statistics in Table 2.

**Table 2:** Mean, standard deviation, minimum, maximum values, normality test statistics (Shapiro-Wilk’s test, skewness and kurtosis) for the dimensions of the scales involved in the study: Work-Family Conflict Questionnaire and Olson-Family Test (FACES IV.) (*N* = 273; scale 1–5)

	Survey dimensions	*N*	M	SD	Min	Max	W	*p*	Skewness	Kurtosis
Work-family Conflict Questionnaire	**work-to-family_conflict**	273	1.696	0.691	1	3.8	0.875	< 0.001	1.00	0.215
	**family-to-work_conflict**		1.404	0.601	1	4	0.715	< 0.001	1.95	3.88
	**work_involvement**		2.262	0.693	1	3.75	0.962	< 0.001	− 0.195	− 0.716
	**family_involvement**		3.728	0.364	2.25	4	0.756	< 0.001	-1.50	1.84
	**job_satisfaction**		3.072	0.789	1	4	0.911	< 0.001	− 0.683	− 0.317
	life_satisfaction		3.112	0.647	1.2	4	0.941	< 0.001	− 0.741	0.256
Olson-Family Test (FACES-IV.)	**cohesion**		3.756	0.354	2.33	4.43	0.922	< 0.001	-1.22	2.16
	**flexibility**		3.516	0.390	1.86	4.48	0.968	< 0.001	-0756	1.58
	**family_communication**		4.161	0.700	1	5	0.893	< 0.001	-1.30	1.94
	**family life satisfaction**		4.035	0.786	1	5	0.893	< 0.001	-1.36	2.38

The respondents were found to be mostly satisfied with their lives. Family involvement is more prevalent among the respondents than work involvement. In terms of the family unit dimensions, the overall responses indicate that the respondents reported well-functioning family units.

To analyse our hypotheses, we first conducted the Shapiro-Wilk test of normality, and since none of the variables are normally distributed, nonparametric tests were used. More specifically, Spearman’s rank correlations were calculated and are shown in Table 3.

In the analysis of our first hypothesis, the Spearman correlation test did not show a significant relationship (*r*=-.017, *p* = .784), so our first hypothesis was not confirmed. In our second hypothesis, where we tested the relationship between the dimensions of work and family involvement, the results confirmed our hypothesis (*r* = − .167, *p* = .006). We did not find a significant relationship between mothers’ involvement in work, the number of children (*r* = .077, *p* = .202) and the age of the youngest child (*r* = .091, *p* = .132), thus our third hypothesis was not confirmed. For our fourth and fifth hypotheses, we investigated the relationship between family cohesion, family flexibility, family life satisfaction, and job satisfaction. Our hypotheses were supported by the results obtained, according to which our last two hypotheses (H4 and H5) were confirmed.

**Table 3:** Spearman correlation test results along the variables

Variable		1	2		4	5	6	7	8	9		11	12	13	14
1 Age of youngest child	r	1.000													
	p	.													
2 Number of children	r	0.064	1.000												
	p	0.296	.												
4 Work-to-family_conflict	r	0.010	0.055	− 0.076	1.000										
	p	0.870	0.362	0.231	.										
5 Family-to-work_conflict	r	− 0.197^**^	0.085	− 0.064	0.470^**^	1.000									
	p	0.001	0.161	0.313	0.000	.									
6 Family_involvement	r	− 0.041	− 0.104	0.180^**^	− 0.205^**^	− 0.166^**^	1.000								
	p	0.500	0.087	0.004	0.001	0.006	.								
7 Work_involvement	r	0.091	0.077	− 0.019	− 0.017	0.082	− 0.167^**^	1.000							
	p	0.132	0.202	0.761	0.784	0.177	0.006	.							
8 Job_satisfaction	r	− 0.088	0.074	0.115	− 0.436^**^	− 0.216^**^	− 0.027	0.349^**^	1.000						
	p	0.147	0.225	0.071	0.000	0.000	0.660	0.000	.						
9 Life_Satisfaction	r	− 0.165^**^	− 0.110	0.458^**^	− 0.300^**^	− 0.204^**^	0.174^**^	− 0.014	0.355^**^	1.000					
	p	0.006	0.069	0.000	0.000	0.001	0.004	0.823	0.000	.					
11 Cohesion	r	− 0.011	− 0.018	0.299^**^	− 0.145^*^	− 0.093	0.176^**^	0.013	0.218^**^	0.338^**^	0.234^**^	1.000			
	p	0.857	0.772	0.000	0.017	0.124	0.003	0.832	0.000	0.000	0.000	.			
12 Flexibility	r	0.064	0.020	0.331^**^	− 0.288^**^	− 0.229^**^	0.155^*^	0.184^**^	0.324^**^	0.432^**^	0.371^**^	0.488^**^	1.000		
	p	0.289	0.740	0.000	0.000	0.000	0.010	0.002	0.000	0.000	0.000	0.000	.		
13 Family_communication	r	0.038	− 0.008	0.436^**^	− 0.281^**^	− 0.253^**^	0.251^**^	− 0.056	0.203^**^	0.440^**^	0.310^**^	0.572^**^	0.627^**^	1.000	
	p	0.528	0.894	0.000	0.000	0.000	0.000	0.358	0.001	0.000	0.000	0.000	0.000	.	
14 Family life satisfaction	r	− 0.023	− 0.045	0.414^**^	− 0.243^**^	− 0.253^**^	0.170^**^	− 0.015	0.247^**^	0.506^**^	0.432^**^	0.528^**^	0.561^**^	0.776^**^	1.000
	p	0.705	0.457	0.000	0.000	0.000	0.005	0.804	0.000	0.000	0.000	0.000	0.000	0.000	.

*Note* *p < .05; **p < .01

There was a strong positive correlation between family cohesion and satisfaction with family life (*r* = .528, *p* = .001), and a positive correlation with job satisfaction (*r* = .218, *p* = .001). A strong positive correlation was observed with life satisfaction for family flexibility (*r* = .562, *p* = .001), and a positive correlation was also found for job satisfaction (*r* = .324, *p* = .001) (Table 3).

Results of H1 did not show any relationship between work-to-family conflict and work involvement, but concerning the possible moderating role of family flexibility between work involvement (commitment) and work-to-family conflict, - according to Olson’s Circumplex Model [12]) - an additional moderation analysis was conducted using Jamovi, the results of which are summarised in Tables 4, 5 and 6. Related to the results of our sixth hypothesis, family flexibility had a moderation effect in the case of work involvement and work-to-family conflict, but not in the case of family involvement and family-to-work conflicts. Work-to-family conflict seems to be connected with work involvement only when family flexibility is low.

**Table 4:** Moderation estimates for moderation model between work involvement and work-to-family conflict using family flexibility as moderator variable

Moderation Estimates				
	Estimate	SE	Z	*p*
Work_involvement	0.039	0.057	0.692	0.489
Family_flexibility	− 0.567	0.101	-5.621	< 0.001
Work_involvement*Family_flexibility	− 0.266	0.132	-2.019	0.044

**Table 5:** Slope estimates for moderation model between work involvement and work-to-family conflict using family flexibility as moderator variable

Simple Slope Estimates				
	Estimate	SE	Z	*p*
Average	0.399	0.058	0.688	0.491
Low (-1SD)	0.144	0.071	2.009	0.045
High (+ 1SD)	0.064	0.083	− 0.771	0.441

**Table 6:** Moderation estimates for moderation model between family involvement and family-to-work conflict using family flexibility as moderator variable

Moderation Estimates				
	Estimate	SE	Z	*p*
Family_involvement	− 0.163	0.099	-1.634	0.102
Family_flexibility	− 0.309	0.090	-3.428	< 0.001
Family_involvement*Family_flexibility	0.259	0.271	0.954	0.340

## Discussion and conclusions

The phenomenon of work-family conflict is part of people’s daily lives, and this problem is increasingly affecting working mothers due to the ever-increasing workload and demands from both directions. There is typically a conflict of interest between involvement in work and family life. A well-functioning family, with adequate partner support and relationship satisfaction, can correlate with the positive outcome of the effort to achieve a balance. Moreover, it can play a key role in balancing childcare and work responsibilities.

In the present study, we wanted to examine the relationships between work-family conflict variables and family and work involvement and structure among working mothers with children. The results suggest that our first hypothesis, that there is a relationship between higher work involvement and the frequency of work-to-family conflict, is not confirmed, contrary to what we expected. The correlation of the two phenomena is formulated in connection with the idea that excessive involvement in work (high number of hours, overtime), and high energy invested in work, take time away from family life and presence, generating conflicts between the two areas [29, 30]. This result may be explained by the size of the sample (*N* = 273), hence it may be worth investigating a larger sample in the future. It may also be explained by the fact that the mothers in the vast majority of cases reported that they were satisfied with their family life, which may act as a protective net against the demands of the workplace [29, 61]. Our second hypothesis confirmed the expected mutual negative relationship i.e., the more involved individuals are in work, the less they are able to “participate” in family life [25, 29]. No significant correlation was found between the number of children, the age of the youngest child, and mother’s involvement in work. This outcome may be based on good working conditions, flexible working hours and the role of social support and a well-functioning family unit, especially in terms of the number of children. Furthermore, the fact that the women in the sample have 2 children on average, followed by mothers with 1 child, is likely to be significant, making it easier to organise work-family activities than those in households with more children. We also examined whether adequate family cohesion and flexibility imply higher levels of family life and job satisfaction. In both cases, we identified a strong positive relationship for family life satisfaction and also a positive relationship for job satisfaction. Related to Olson’s Circumplex model [12], for our hypothesis 6, we deemed it important to examine whether family resilience would show a moderating effect on relation work/family involvement and work-to-family conflict and family-to-work conflict. The results seem to suggest that a moderating effect only holds for work involvement. A positive relationship between work-to-family conflict and work involvement appears only when family resilience is low.

The work-family issue is very complex, maintaining a balance is a difficult task, over-involvement in one area can be a hindrance, investing extra energy can become a hindrance to being “present” in the other area, which can be a source of serious conflicts in the lives of individuals. This kind of “double burden” of meeting demands and expectations is presumably more difficult for mothers, based on the assumption that even today, due to traditional gender roles, women are more involved in the family than men [59]. However, the international literature is full of recent studies that have examined various psychological aspects of work-life balance. These studies relate the issue to models such as perceived stress, mental state and social organisation, i.e. the extent to which working mothers are supported at a societal level and how they are supported. In their study, Prickett, Crosnoe et al. [62] report that mothers of young children in their study had better physical and mental health outcomes if they worked in workplaces where more socio-emotional resources were available. The results of a survey by Brenning, Mabbe et al. [63] highlight the importance of work-life balance, both in terms of parents’ mental health (parents’ emotional exhaustion) and the quality of parenting. Regarding mental health, Zhang et al. [64] also analysed stress, perceived stress and exhaustion in the context of work-life balance. Following this line of thinking, Luhr, Schneider et al. [65] also examined stress as a consequence of suboptimal job characteristics and role conflict (work, private life, childcare). Given the results of the present study, especially the number of children and the age of the youngest child, and the orientations of recent research, it would be worthwhile to explore this issue in a more complex study with a larger and more representative sample in the future. In further research, it is also worth taking into account the economic aspect - for example, the number of earnings, the number of days off from work due to a child’s illness - its importance for job satisfaction, women’s sense of well-being, career plans and more.

### Limitations

Our study has limitations. The sample we could reach is not representative due to its specific nature and the sampling method. The sample size was severely limited by the willingness to complete, as it was difficult to reach respondents. We also must mention that the reliability of the questionnaire is low in some scales which can be caused by the population or the sample itself. Also, the changes that occurred since the original questionnaire was validated could lead to the inappropriate reliability. The reliability value of family involvement scale was low, and questionable. Results with this scale must be interpreted carefully, and investigation is needed in future studies to understand the lower value. The analyses of this pilot study do not allow conclusions about cause-and-effect relationships due to the correlational nature of the research. For this reason, we have to emphasise that our data cannot be generalised for the population. Furthermore, even though that most of the research on this issue focuses on working mothers, we consider it important to include fathers in our future extended research, as men, fathers, are increasingly becoming involved in family life due to changes in parental roles [66–68].

### Electronic supplementary material

Below is the link to the electronic supplementary material.

Supplementary Material 1

## Data Availability

The datasets generated and/or analyzed during the current study are not publicly available because part of an ongoing PhD thesis and an application project.
