# Supplementary material for: Relationships between work-family conflict and family structure in the lives of working mothers in Hungary – a pilot study
Source: BMC Psychol. 2024 Aug 6;12:427. doi: 10.1186/s40359-024-01925-0 (PMC11304997; doi:10.1186/s40359-024-01925-0)
Supplement: Supplementary file 1 — Supplementary Material 1 [file 40359_2024_1925_MOESM1_ESM.docx]

**DEMOGRAPHIC QUESTIONNAIRE**

1. **DEMOGRAPHIC DATA**
2. **Age (Please write on the dotted line)** ........ year
3. **Place of residence**

Capital city
Chief town of a county
City
Municipality
Village

1. **Highest level of education (per respondent)**Less than 8 years of primary education
   8 years of primary education
   Vocational school, technical school
   secondary school
   higher education (college, university)
   other: ……………………………
2. **Your partner's highest level of education (if currently in a relationship)**
3. less than primary school

8 years of primary education
vocational school, technical school
secondary school
higher education (college, university)
other: ……………………………

1. **How many children do you have?**One
   Two
   Three
   Four
   Five
   Six or more
2. **How old is/are your child/children? (Please write the age(s) in ascending order with numbers on the dotted line)** ……………………………………………………
3. **What is your current relationship?**Single
   Intimate relationship
   Registered partnership
   Married
   Divorced
   Widowed
4. **WORK CHARACTERISTICS**
5. **What is your current occupation? (Please write on the dotted line.)** ……………………………………
6. **What is your current job title? (You can tick more than one of the options listed)**

Trainee

Government employee

Employee (physical staff)

Employee (intellectual staff)

Middle management

Senior Management

Entrepreneur

1. **How long have you been in your current job?**

Less than 1 year
More than 1 year but less than 5 years
More than 5 years
Currently unemployed

1. **Which of the following statements is typical of your job and working hours?**

- I work in Hungary, my working hours are family-friendly (e.g. my working hours/schedule can be adapted to family life)
- I work in Hungary, but my work schedule is not family-friendly (e.g. I am away relatively often or my working hours/schedule mean that we have less time together)
- I work abroad and am away from home for weeks at a time

1. **Which of the following statements is typical of your partner's job and working hours? (If currently in a relationship)**

- He/she works in Hungary and his/her working hours are family-friendly (e.g.: his/her working hours/schedule can be adapted to family life)
- He/she works in Hungary, but his/her working hours are not family-friendly (e.g. he/she is away relatively often or his/her working hours/schedule means that they can see each other less often)
- He/she works abroad and is away from home for weeks at a time

1. **Please tick the statement/s typical for your working schedule. You can underline more than one of the options listed.)**

I work shifts
I work part-time
full-time but not shift work
flexible working hours
I work modified hours due to the effects of pandemic COVID-19 (e.g. partial home office, full home office, other)

1. **Which of the following statements most affects you in your current job? (You can underline more than one of the options listed.)**

Financial resources
Professional development
Language development
Working conditions
Lack of job opportunities at home
Financial basis for the future
Moving the whole family abroad in the future
Appointment/career progression
Other

1. **How satisfied are you with your family's financial situation? (Please circle on a scale of 1 to 5 below, where 1 - I am very dissatisfied; 5 - I am completely satisfied)**

1 2 3 4 5
